# ECMO-treatment in patients with acute lung failure, cardiogenic, and septic shock: mortality and ECMO-learning curve over a 6-year period

**DOI:** 10.1186/s40560-018-0352-2

**Published:** 2018-12-18

**Authors:** Norbert Banjas, Hans-Bernd Hopf, Ernst Hanisch, Benjamin Friedrichson, Julia Fichte, Alexander Buia

**Affiliations:** 1 Department of Visceral and Thoracic Surgery, Asklepios Klinik Langen, Röntgenstr 20, 63220 Langen, Germany; 2 Department of Anaesthesia and Peri-operative Medicine, Asklepios Klinik Langen, Röntgenstr 20, 63220 Langen, Germany

**Keywords:** Extracorporeal membrane oxygenation, CUSUM-learning curve, Mortality, Predictors, ARDS, Cardiogenic shock, Septic shock

## Abstract

**Background:**

Based on promising results over the past 10 years, the method of extracorporeal membrane oxygenation (ECMO) has developed from being used as a ‘rescue therapy’ to become an accepted treatment option for patients with acute lung failure (ARDS). Subsequently, the indication was extended also to patients suffering from cardiogenic and septic shock. Our aim was to evaluate hospital mortality and associated prognostic variables in patients with lung failure, cardiogenic, and septic shock undergoing ECMO. Furthermore, a cumulative sum (CUSUM) analysis was used to assess the learning curve of ECMO-treatment in our department.

**Methods:**

We retrospectively analysed the data of 131 patients undergoing ECMO treatment in the intensive care unit of the Asklepios Hospital of Langen over the time period from April 2011 to July 2016. We categorised the patients into three groups: lung failure (*n* = 54); cardiogenic shock (*n* = 58); and septic shock (*n* = 19). The primary outcome variable was hospital mortality along with identification of prognostic variables on mortality before initiating ECMO using logistic regression. Second outcome variable was the learning curve of our department in patients with ECMO.

**Results:**

6-year hospital mortality was 54% in patients with lung failure, 59% in patients with cardiogenic shock, and 58% in patients with septic shock.

The CUSUM analysis revealed a typical learning curve with a point of inflection in the year 2014. Patients treated before 2014 had a worse outcome (*p* = 0.04 whole cohort; *p* = 0.03 for lung failure). Furthermore, less than 20 treatments per year respectively treatment before 2014 were associated negatively with hospital mortality of lung failure patients showing an odds ratio of 4.04, as well as in the entire cohort with an odds ratio of 3.19.

**Conclusion:**

For the first time, a steep ECMO-learning curve using the CUSUM tool has been described. Obviously, the experience with ECMO has to be taken into account when defining the role of ECMO in ARDS, cardiogenic, and septic shock.

## Background

In addition to its application as veno-venous (vv-) ECMO in cases of severe lung failure, ECMO is now accepted as a treatment option in the form of veno-arterial (va) or veno-venous-arterial (vva) ECMO for patients with cardiogenic shock [1]. Current guidelines on respiratory failure and cardiogenic shock in the context of myocardial infarction now recommend ECMO treatment as a ‘rescue therapy’ for severe cases [2, 3], even if, as in the case of patients with cardiogenic shock, there is still only limited evidence for its effectiveness [2, 4]. In fact, the number of veno-arterial and veno-venous ECMO treatments over the last few years has risen substantially both in Germany and around the world [5, 6]. Whilst the use of ECMO in patients with ARDS or cardiogenic shock at least as rescue therapy is an accepted approach, little data are available regarding its use in adult patients suffering from septic shock, with survival rates varying between 15% and 71% [7–10].

Accordingly, our first objective was to assess hospital mortality in our patients with acute lung failure, cardiogenic, and septic shock treated by ECMO and to identify pre-ECMO variables associated with good or poor outcome respectively, i.e. which patients probably might benefit from ECMO and which not. Second objective was to evaluate whether in a complex medical procedure like ECMO a ‘learning curve’ can be observed with increasing routine and patients per year.

## Methods

### Study design

We analysed the data of 131 patients in a retrospective cohort study from April 2011 to July 2016, treated with a veno-venous ECMO (vv-ECMO), veno-arterial ECMO (va-ECMO) or veno-venous-arterial ECMO (vva-ECMO) in the Asklepios Hospital Langen. We divided our patients in three indication groups: acute lung failure, cardiogenic, and septic shock. The presentation of the study has been carried out following STROBE guidelines (Strengthening the Reporting of Observational Studies in Epidemiology) [11]. The study was approved by the Ethics Commission of the State Medical Council of Hessen (file reference no. 88/2017).

### Setting

The Asklepios Hospital in Langen is a teaching hospital of the Goethe University in Frankfurt am Main with 10 departments, 401 beds, and > 16,000 in-patients per year. ECMO treatment is carried out on the interdisciplinary intensive care unit, led by anesthesiology, with 14 artificial-respiration beds, equipped with haemodiafiltration and dialysis treatment options. Data collection was carried out between November 2017 and January 2018.

The ECMO treatment team made all decisions regarding treatment with ECMO. Where respiratory insufficiency was the sole indication, the treatment was carried out as vv-ECMO. In patients with septic or cardiogenic shock, the treatment was carried out as va-ECMO or vva-ECMO. Cannulations were guided by ultrasound imaging. In the vv-ECMO variant, cannulation was carried out in the right internal jugular vein (15 or 19F) and the femoral vein (23 or 25F), or in cases of AVALON double-lumen cannulas (31F) via the right internal jugular vein alone. In the va-ECMO mode, cannulation was carried out in the femoral artery (15 or 17F) and vein (23 or 25F). When applying the vva-ECMO mode, an additional venous cannula was placed in the right internal jugular vein in addition to the cannulas in the femoral artery and vein (15 or 19F).

### Study participants and study size

We identified 132 patients being treated with ECMO (vv-ECMO, va-ECMO, vva-ECMO) on the intensive care unit of the Asklepios Hospital in Langen. These patients were treated consecutively over the aforementioned time period. Of these, 131 were included in the study: one patient was excluded from the study due to lacking data. We divided patients into three groups:

Group 1 (lung failure) included all patients that received veno-venous ECMO for hypoxic and/or hypercapnic respiratory failure, regardless of the underlying disease. The indication for use of ECMO was based on the Berlin definition of acute lung failure [12] and was determined after having exhausted all conventional treatment options. Patients simultaneously suffering from treatment-refractory septic shock and treated with vva-ECMO were not included in this group but included in group 3 considering the septic shock to be the primary disease.

Group 2 (cardiogenic shock) included all patients that received va-ECMO treatment for cardiogenic shock. Post-cardiotomy patients were excluded. Cardiogenic shock was defined as persistent hypotension despite adequate volume replacement (ultrasound of inferior vena cava) and the need for noradrenaline infusion at a dose > 0.5μg/kg/min in order to maintain a mean arterial blood pressure ≥ 65 mmHg.

Group 3 (septic shock) included all patients treated with vva-EMCO to provide both circulatory and respiratory support due to suffering from treatment-refractory septic shock. The definition of septic shock corresponds with the third international consensus definitions for sepsis and septic shock [13]. Criteria for application of vva-ECMO were mean arterial pressure < 65 mmHg and/or progressive lactic acidosis and end-organ dysfunction despite adequate administration of fluids and increasing noradrenaline infusion (> 0.5μg/kg/min).

### Data sources and variables

After identification of patients, the following variables were recorded: age, gender, weight, diagnosis and pre-existing conditions, blood gas analyses (pH, pO2, and pCO2, base deficit, lactate), need for resuscitation, Simplified Acute Physiology Score (SAPS) II score, Sequential Organ Failure Assessment (SOFA) score, length of hospital and ICU stay, duration of artificial ventilation, need for and number of patients treated with dialysis, and necessity of tracheostomy. Scores and laboratory variables were recorded at comparable times. All the data collected were recorded in a Microsoft Excel database. The primary outcome variable was the hospital mortality in the individual groups along with the identification of prognostic pre-ECMO variables on mortality. Second outcome variable was to assess whether there was a learning curve of the procedure with increasing routine and patient numbers over time.

### Statistics

A cumulative sum chart (CUSUM chart) was used to investigate a possible learning curve. We used the non-risk-adjusted cumulative observed minus expected failure graph procedure, such as that described by Rogers et al. [14]. The CUSUM curve is calculated on the basis of the following considerations: where the ECMO treatment is successful, *X*_*i*_ = 0; and where the treatment is not successful, and the patient dies over the period of the hospital stay, *X*_*i*_ = 1. On the basis of epidemiological data from Karagiannidis et al. [5], we selected an acceptable error rate for ECMO treatment in Germany, which corresponds to the expected mortality of *p*_0_ = 0.6 for a mixed vv and va-ECMO population. The formula for calculation of the CUSUM curve was calculated as follows: *C*_*i*_ = *C*_(*i* − 1)_ + (*X*_*i*_ − *p*_0_) with *C*_0_ = 0. The interpretation of the curve characteristics is carried out as follows: each successful treatment lowers the CUSUM value by *p*_*0*_, and the curve drops. Each failed treatment results in an increase of 1−*p*_*0*_, and the curve rises. Where the actual error rate corresponds to the rate expected, the curve will oscillate along a horizontal axis. Logistic regression was used for identification of variables influencing mortality, specifying the odds ratio (OR) and the confidence interval (CI). Logistic regression was first performed as a univariate analysis. Variables with a significant *p* value in the univariant analysis were furthermore used for multivariate logistic regression as an additional sensitivity analysis. Due to the small sample size, multivariate analysis was performed in the entire patients and stepwise backwards elimination process was used with *p* <  0.05.

The statistical analysis was performed with BiAS (version 11.08, Epsilon-Verlag). The null hypothesis was that there were no differences between the three groups in the recorded variables. The normal distribution verification was carried out using the Kolmogorov-Smirnov-Liliefors test. Quantitative data were specified as median values with interquartile range; nominal data were specified as a frequency *n* (%). Differences in medians of quantitative data were analysed with the Kruskal-Wallis test. Differences in categorical variables were tested using the Mantel-Haenszel test or the *χ*^*2*^ test. The null hypothesis was rejected with a two-tailed *p* value < 0.05.

## Results

### Demographic data of the patients

We investigated 131 patients who received ECMO treatment from April 2011 to July 2016. Patients were categorised as follows: group 1 (lung failure) contained 54 patients; group 2 (cardiogenic shock) contained 58 patients; and group 3 (septic shock) contained 19 patients.

Table 1 gives an overview of the demographic and clinical data of the three groups, as well as the distribution of cases before 2014. Table 2 shows the underlying diseases for patients in groups 1–3.

**Table 1:** Patients and treatment characteristics

	RF, *n* = 54	CS, *n* = 58	SS, *n* = 19	*p* value
Age, years	62 (53–70)	67 (55–73)	62 (55–73)	0.28
Weigh, kg^a^	76.5 (64–102)	88 (70–99)	89.5 (77–102)	0.33
Male	29 (54)	37 (64)	14 (74)	0.26
SOFA^b^	6 (3–9)	10 (8–11)	8 (6–12)	< 0.01
SAPS II^c^	40 (31–60)	67 (48–78)	42 (32–61)	< 0.01
Comorbidities				
-Art. hypertension	33 (61)	34 (60)	8 (42)	0.33
-Coronary disease	7 (13)	40 (70)	4 (21)	< 0.01
-Diabetes mellitus	11 (20)	16 (28)	2 (11)	0.26
-Hyperlipidaemia	4 (7)	8 (14)	5 (26)	0.11
-Kidney injury	7 (13)	2 (4)	1 (5)	0.16
-COPD	26 (48)	3 (5)	4 (21)	< 0.01
Duration of hospitalisation, days	27 (15–40)	10 (4–36)	26 (18–67)	< 0.01
Duration of ICU stay, days	20 (8–31)	7 (3–25)	20 (17–65)	< 0.01
Time on ECMO support, days	12 (5–19)	5 (3–8)	14 (9–25)	< 0.01
CPR pre-ECMO	3 (6)	38 (66)	3 (16)	< 0.01
Mechanical ventilation time, h	391 (122–687)	129 (42–413)	423 (304–772)	< 0.01
Renal replacement therapy	36 (67)	45 (78)	18 (95)	< 0.05
Tracheotomy	33 (61)	20 (34)	17 (89)	< 0.01
Weaning from ECMO	35 (65)	30 (52)	10 (53)	0.34
Death	29 (54)	34 (59)	11 (58)	0.86
ECMO-treatment before 2014	27 (50)	17 (29)	1 (5)	< 0.01

Data is given as median (interquartile range) and *n*(%)

*RF*, respiratory failure; *CS*, cardiogenic shock; *SS*, septic shock; *SOFA*, Sequential Organ Failure Assessment; *SAPS II*, Simplified Acute Physiology Score; *COPD*, chronic obstructive lung disease; *CPR*, cardiopulmonary resuscitation

^a^(*n* = 48 in RF, *n* = 46 in CS)

^b^(*n* = 50 in RF, *n* = 56 in CS)

^c^(*n* = 51 in RF, *n* = 56 in CS)

**Table 2:** Aetiology of respiratory failure, cardiogenic shock and septic shock

	RF, *n* = 54	CS, *n* = 57	SS, *n* = 19	*p* value
Pneumonia	31 (57.41)	–	10 (52.63)	0.41
Intra-abdominal infection	4 (7.41)	–	8 (42.11)	< 0.01
Soft tissue infection	1 (1.85)	–	1 (5.26)	0.49
Trauma	1 (1.85)	–	–	–
Cardiac decompensation	6 (11.11)	–	–	–
Shock	3 (5.56)	–	–	–
Decompensated chronic lung disease	3 (5.56)	–	–	–
Other	5 (9.25)	–	–	–
Acute myocardial infarction	–	41 (70.69)	–	–
Cardiomyopathy	–	4 (6.9)	–	–
Pulseless electrical activity	–	4 (6.9)	–	–
Pulmonary emboli	–	3 (5.17)	–	–
Ventricular fibrillation	–	2 (3.45)	–	–
Myocarditis	–	1 (1.72)	–	–
Other	–	3 (5.17)	–	–

Data is given as *n*(%)

*RF*, respiratory failure; *CS*, cardiogenic shock; *SS*, septic shock

In group 1, the most common cause of lung failure was an infection of the lungs (*n* = 31, 57%). The median SOFA score before starting ECMO was 6; the median SAPS II score 40.

In group 2, the most common cause for cardiogenic shock was myocardial infarction (*n* = 40, 69%). The median SOFA score before starting ECMO was 10; the median SAPS II score 67. The patients in group 2 had the highest SAPS II score when compared with patients from group 1 and group 3 (*p* <  0.01 vs. group 1; and *p* = 0.03 vs. group 3) and the highest SOFA score when compared with patient from group 1 (*p* <  0.01).

In group 3, the most common cause of septic shock was infection of the lungs (*n* = 10, 53%). The second common cause was an abdominal infection (*n* = 8). The causes of abdominal septic shock were anastomotic insufficiency (*n* = 3), pancreatitis (*n* = 2), gastric perforation (*n* = 1), mesenteric vein thrombosis (*n* = 1), and cholangitis (*n* = 1). The median SOFA score was 8; the median SAPS II score 42.

### Clinical outcomes

Clinical outcomes are also shown in Table 1. The hospital stays for group 1 (27 days) and group 3 (26 days) were significantly longer than that of group 2 (10 days) (group 1 vs group 2: *p* = 0.01; and group 2 vs group 3: *p* = 0.02) (Table 1). The median duration of ECMO treatment was 11 days for group 1, 5 days for group 2, and 14 days for group 3 (group 2 vs groups 1 and 3 *p* <  0.01). Cardiopulmonary resuscitation (CPR) was carried out previously to the ECMO implantation (within a 24-h time frame) in 3 patients in group 1, 38 patients in group 2, and 3 patients in group 3. A bypass operation was carried out in five patients with cardiogenic shock (group 2). In six out of eight patients with septic shock due to abdominal cause abdominal surgery was performed.

Table 3 shows the blood gas analyses both before and 1 day after implantation of the extracorporeal circulation. The pH value significantly improved and showed standard values after 1 day of ECMO treatment. Also, all other values improved within the first 24 h.

**Table 3:** Blood gas analysis pre ECMO and 1 day after ECMO initiation

Pre-ECMO	RF, *n* = 46	CS, *n* = 49	SS, *n* = 17	*p* value
pH^a^	7.25 (7.18–7.35)	7.11 (6.95–7.25)	7.21 (7.11–7.3)	< 0.01
pCO2, mmHG	67 (54–83)	52 (42–68)	64 (53–84)	< 0.01
BE, mmol/l	2 (− 5 to 8)	−14 (− 21 to − 5)	−4 (− 8 to − 1)	< 0.01
Lactate, mg/dl	13 (8–26)	73 (34–114)	29 (13–62)	< 0.01
pO2/FiO2^b^	145 (99–233)	158 (83–254)	111 (77–147)	0.23
1 day after ECMO initiation	RF *n* = 48	CS *n* = 44	SS, *n* = 19	*p* value
pH, mmHg	7.40 (7.35–7.46)	7.39 (7.34–7.44)	7.42 (7.38–7.49)	0.43
pCO2, mmHg	48 (43–52)	42 (40–45)	47 (44–49)	< 0.01
BE, mmol/l	6 (2 to 9)	1 (−1 to 3)	4 (2 to10)	< 0.01
Lactate, mg/dl	13 (9–21)	22 (16–34)	17 (14–35)	< 0.01
pO2/FiO2	199 (163–262)	278 (180–472)	168 (117–260)	< 0.01

Data is given as median (interquartile range)

*RF*, respiratory failure; *CS*, cardiogenic shock; *SS*, septic shock; *BE*, base excess

^a^(*n* = 47 in RF)

^b^(*n* = 47 in RF, *n* = 43 in CS, *n* = 16 in SS)

### Mortality

The 6-year hospital mortality for the entire study population was 56%. In group 1, 29 of 54 patients died (54%), in group 2, 34 of 58 (59%), in group 3, 11 of 19 (58%) (Table 1). Patients with septic shock due to abdominal infection compared with lung infection had the worst prognosis: six out of eight patients died. Patients with septic shock due to lung infection survived in 5 of 10 cases. The patient with the soft tissue infection survived. Withdrawal of ECMO was successful in 35 of 54 cases in group 1, in 30 of 58 cases in group 2, and 10 of 19 cases in group 3.

Since national guidelines for the treatment of respiratory insufficiency recommended at least 20 ECMO treatments per year [2], we investigated how many patients have been treated by us each year: in the years from 2011 to 2016, we treated 10, 16, 19, 27, 32, and 27 patients respectively.

We subsequently examined how many patients had died in the 3-year period with < 20 patients per year. In fact, in the period 2011–2013, 31 of the 45 patients treated died (69%), whereas in the period 2014 to 2016, only 43 of 86 patients treated died (50%) (*p* = 0.04). With respect to the groups 1–3, this effect was also apparent in groups 1 and 2, although not significant in group 2. In the period 2011–2013, 19 of 27 patients from group 1 died (70%), whereas in the period 2014–2016, only 10 of 27 patients died (37%) (*p* = 0.03).

In group 2, 12 of 17 patients (71%) died in the time period from 2011 to 2013, whereas in the 2014–2016 time period, 22 of 41 patients died (54%) (*p* = 0.37).

For the septic shock group, no comparison could be made since most of the patients with septic shock were treated in the period 2014–2016.

**Fig. 1:**
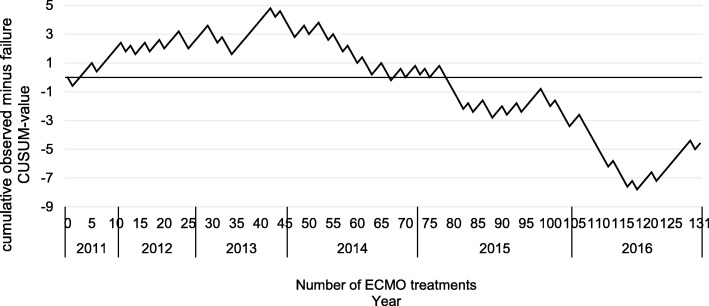
Cumulative observed minus failure (CUSUM) chart for in-hospital mortality after ECMO treatment. Legend: the vertical axis shows the CUSUM value, which increases for each failure or decreases on each success. The horizontal axis shows the number of ECMO treatments and the corresponding year. Expected failure rate was set at the expected mortality rate of 60% based on the data of the epidemiologic study of Karagiannidis et al. for a mixed vv- and va-ECMO population in Germany [5]. An upward slope represents a failure rate higher than expected and a downward slope represents a failure rate fewer than expected. With a failure rate equal the expected failure rate, the CUSUM curve should oscillate around a horizontal axis

Figure 1 shows our CUSUM learning curve. Mortality rate initially increased followed by a horizontal progression, relating to a mortality rate which corresponds with the expected mortality rate *p*_0_ = 0.6, in addition to a final section, with a falling curve indicating the learning effect with a reduced mortality rate. This downward trend of the curve begins in the year 2014.

Table 4 shows the results from the univariate logistic regression analysis of the variables recorded before application of ECMO with respect to hospital mortality.

**Table 4:** Univariate logistic regression of predictors for in-hospital mortality

	RF		CS		SS	
	Odds ratio (CI)	*p*	Odds ratio (CI)	*p*	Odds ratio (CI)	*p*
Age^a^	1.07 (1.01–1.12)	0.01	1.08 (1.02–1.13)	0.01	1.04 (0.96–1.13)	0.30
Year^a^	0.65 (0.45–0.93)	0.02	0.88 (0.59–1.31)	0.51	2.28 (0.62–8.42)	0.22
< 20 patients/years^a^	4.04 (1.27–12.86)	0.02	2.07 (0.61–7.1)	0.25	NA	NA
SAPS II^b^	1 (0.97–1.03)	0.93	1.03 (1–1.06)	0.03	0.99 (0.94–1.04)	0.71
pH pre-ECMO^c^	0.55 (0.01–50.19)	0.79	0.22 (0–0.66)	0.03	0.21 (0–1118)	0.72
Base excess pre-ECMO^d^	0.94 (0,86–1.03)	0.17	0.89 (0.82–0.97)	0.01	1.04 (0.88–1.22)	0.69
Lactate pre-ECMO^e^	1.02 (0.99–1.05)	0.27	1.03 (1.01–1.05)	< 0.01	0.99 (0.95–1.02)	0.34
Hyperlipidaemia^f^	2.77 (0.26–29.72)	0.40	0.07 (0.01–0.64)	0.02	0.37 (0.04–3.4)	0.38

*RF*, respiratory failure; *CS*, cardiogenic shock; *SS*, septic shock; *SAPS II*, Simplified Acute Physiology Score; *CI*, confidence interval

^a^(*n* = 54 in RF, *n* = 58 in CS, *n* = 19 in SS)

^b^(*n* = 51 in RF, *n* = 56 in CS, *n* = 19 in SS)

^c^(*n* = 47 in RF, *n* = 49 in CS, *n* = 17 in SS)

^d^(*n* = 46 in RF, *n* = 49 in CS, *n* = 17 in SS)

^e^(*n* = 46 in RF, *n* = 49 in CS, *n* = 17 in SS)

^f^(*n* = 54 in RF, *n* = 57 in CS, *n* = 19 in SS)

In patients with lung failure, age (OR 1.07, CI 1.01–1.12; *p* = 0.01), number of patients treated per year (< 20/a) (OR 4.04; CI 1.27–12.86; *p* = 0.02), and the year of performing ECMO (OR 0.65; CI 0.45–0.93; *p* = 0.02) were significantly associated with hospital mortality. In patients with cardiogenic shock, age (OR 1.08; CI 1.02–1.13; *p* = < 0.01), SAPS II score (OR 1.03; CI 1–1.06; *p* = 0.03), pH value (OR 0.22; CI 0–0.66; *p* = 0.03), serum lactate value (OR 1.03; CI 1.01–1.05; *p* = < 0.01), base excess (OR 0.89; CI 0.82–0.97; *p* = 0.01), and hyperlipidaemia (OR 0.07; CI 0.01–0.64; *p* = 0.02) were associated with hospital mortality.

In patients with septic shock, no variables could be identified that were associated with an increased hospital mortality.

Table 5 shows the multivariate logistic regression analysis results of pre-ECMO variables associated with hospital mortality. In the multivariate logistic regression after backward elimination with *p* <  0.05, three variables remained statistically significant on hospital mortality in the whole patients’ population; age (OR 1.08; CI 1.04–1.12; *p* <  0.01), less than 20 patients per year (OR 3.19; CI 1.19–8.51; *p* = 0.02) and pre-ECMO lactate (OR 1.01; CI 1–1.02; *p* = 0.03) were negatively associated with hospital mortality.

**Table 5:** Multivariate logistic regression of predictors for in-hospital mortality in entire patients

	Univariate logistic regression		Multivariate logistic regression	
	Odds ratio (CI)	*p*	Odds ratio (CI)	*p*
Age	1.07 (1.03–1.1)	< 0.01	1.08 (1.04–1.12)	< 0.01
< 20 patients/years	2.21 (1.03–4.76)	0.04	3.19 (1.19–8.51)	0.02
Base excess pre-ECMO	0.96 (0.92–1)	0.03		
Lactate pre-ECMO	1.01 (1–1.02)	0.01	1.01 (1–1.02)	0.03

Univariate logistic regression was performed in entire patients (*n* = 131) except for base excess pre-ECMO and lactate pre-ECMO (*n* = 112)

## Discussion

Our data in patients treated with ECMO showed an overall 6-year hospital mortality of 54% for patients with lung failure, 59% in patients with cardiogenic shock and 58% for patients in septic shock. We were able to show that there is a typical learning curve for the use of the ECMO in our department (Fig. 1). The CUSUM analysis furthermore supports the assumption of a case number-dependent survival rate.

The hospital mortality of patients with lung failure ranges from 39 to 70% [5, 15–19]. Of note, the median average age in 4 of the 6 studies was 44 years, and as such was almost 18 years less than in our study. Thus, the patient’s age seems to be a significant negative prognostic variable on hospital mortality and may explain the mortality differences between these studies and our data. In fact, both the 2013 and 2017 ELSO guidelines specified an increased age as a relative contraindication for both vv-ECMO as well as va-ECMO, even though no specific cut-off age has been stated [1, 20]. A major difference to other studies seems to be the condition of the patients prior to the ECMO treatment. The median SOFA score of patients published previously was 12 [16–18], whereas this score was 6 points for the patients investigated in the present study. One reason for this could be that the decision for treatment was carried out at an early stage in our study. The comparatively high median PaO2/FiO2 ratio before ECMO treatment in our patients, compared with a PaO2/FiO2 ratio < 100 mmHg in the case of the aforementioned studies, can be explained by the fact that in our patients with lung failure, 26 patients exhibited hypercapnic lung failure but only a moderate oxygenation impairment (Horowitz quotient > 150 mmHg).

In our patients with cardiogenic shock, the actual hospital mortality (59%) was considerably lower than the predicted mortality (75%) [21], suggesting a substantial survival benefit of va-ECMO as has been shown in comparable studies [22–26]. The duration of ECMO administration in patients with cardiogenic shock was significantly shorter than in patients with lung failure or patients with septic shock, which might be explained by a rapid stabilisation of the patient by ECMO-treatment. In fact, within the first 24 h, the serum lactate levels (as a marker of tissue perfusion) decreased significantly [27]. Additionally, in comparable studies of patients with cardiogenic shock, the duration of treatment also was only a few days [22, 24], which can in all likelihood be explained by a rapid recovery of the pumping function of the heart, accompanied by an improvement in end-organ perfusion. Consistent with these findings, serum lactate and pH values in our study were variables associated with hospital mortality. This correlation has also been established in other studies [23, 26, 28].

Hospital mortality in septic shock patients treated with va-ECMO is heterogeneous. Published data show in-hospital mortality rates of 78–85% [7, 9], which is higher compared to the hospital mortality of 58% in the present study. There are exceptions: Bréchot et al. performed av-ECMO in patients with septic shock resulting in a hospital mortality rate of only 29% [10]. Since increasing age is negatively associated with survival, the result of Brechot et al. might be explained by the median age of 45 years, significantly lower than the age of patients from other studies, including our study. The extension to vva-ECMO mode was administered in cases of patients with septic shock and ARDS to achieve both efficient oxygenation as well as sufficient haemodynamic support. For example, Yeo et al. performed vva-ECMO in eight patients with ARDS and septic shock (mean age 51 years) of whom four survived, i. e., a mortality rate similar to our results [8]. Since severe sepsis/septic shock are often associated with acute lung failure [29], vva-ECMO might have an advantage over va-ECMO. However, this concept has to be proven yet.

We found a clear relationship between the number of treated patients and the survival rate. However, we cannot distinguish some clear-cut reasons for this finding. National guidelines for the treatment of respiratory insufficiency published in 2016 recommend that centres providing ECMO therapy should treat at least 20 patients per year [2]. In our patients, hospital mortality in the period 2014–2016 fell with > 20 ECMO treatments per year from 69% to 50%; in patients with lung failure, this fall was even more pronounced: 70% to 37%. Consistent with this, the CUSUM curve shows a downward trend (after an initial rise and short horizontal phase), starting in 2014. This downward trend reflects a lower mortality rate than expected, suggesting that a minimum number of ECMO treatments per year is a prerequisite for successful ECMO treatment. Furthermore, less than 20 treatments per year respectively the treatment before 2014 were associated negatively with hospital mortality of lung failure patients showing an odds ratio of 4.04 (Table 4) and in the entire cohort with an odds ratio of 3.19 (Table 5). This case number-dependent probability of survival in patients treated with ECMO can also be found in the ELSO registry. Centres with more than 30 patients per year exhibit significantly lower death rates than centres with a number of cases per year of 5 or less [30]. Finally, in the CESAR study, all ARDS patients were transferred to a specialised centre with greater experience in the ECMO treatment [19], and as such, the high number of cases performed by this hospital may have contributed to the better survival rate.

Our investigation has limitations. First, it is a single-centre study with a relatively small number of cases and a retrospective study design. Second, the decision to apply an ECMO therapy is made on a case-by-case basis by the treating doctor. Third, a standardised protocol defining when an ECMO therapy indication applies does not exist, which may lead to selection bias. This also applies for the time of implantation, which is decided solely on the basis of the treating physician’s best judgement. Fourth, our inhomogeneous cohort due to a wide range of underlying diseases may limit the significance of our findings. Finally, it should be noted that in 29% of patients, there are some missing data, mostly blood gas analysis results, which also limit the conclusiveness of the study.

## Conclusion

Our study has shown that in patients with acute lung failure, cardiogenic, and septic shock undergoing ECMO treatment, there are three major variables associated with hospital mortality: age, pre-ECMO lactate, and experience with the procedure. The CUSUM analysis shows that there is a case- and time-dependent learning curve for the usage of ECMO therapy. In our hospital, it took about 3 years and about 50 treatments to get a change in the gradient of the learning curve. Accordingly, one factor determining outcome in patients with ECMO is obviously the experience of the ECMO centre.

## Abbreviations

ARDS: Adult respiratory distress syndrome
CI: Confidence interval
CUSUM: Cumulative Sum
ECMO: Extracorporeal membrane oxygenation
OR: Odds ratio
SAPS: Simplified Acute Physiology Score
SOFA: Sequential Organ Failure Assessment
