# Correction to: ECMO-treatment in patients with acute lung failure, cardiogenic, and septic shock: mortality and ECMO-learning curve over a 6-year period

**DOI:** 10.1186/s40560-019-0362-8

**Published:** 2019-01-31

**Authors:** Norbert Banjas, Hans-Bernd Hopf, Ernst Hanisch, Benjamin Friedrichson, Julia Fichte, Alexander Buia

**Affiliations:** 1 Department of Visceral and Thoracic Surgery, Asklepios Klinik Langen, Röntgenstr 20, 63220 Langen, Germany; 2 Department of Anaesthesia and Peri-operative Medicine, Asklepios Klinik Langen, Röntgenstr 20, 63220 Langen, Germany

**Correction: J Intensive Care**

**https://doi.org/10.1186/s40560-018-0352-2**

In the original publication of this article [1], an Author’s Note section is missing in Declaration. The content of this section is below:

Some data concerning specifically ECMO-treatment in patients with septic shock have been published previously in a German journal with respect to a completely different research question: Friedrichson B, Fichte J, Banjas N, Schütz M, Hopf H-B. Extrakorporale Membranoxygenierung bei erwachsenen Patienten mit septischem Schock. Anästhesie und Intensivmed. 2018;59:698–704.
